# Effect of Pulsed Magnetic Field on the Microstructure of QAl9-4 Aluminium Bronze and Its Mechanism

**DOI:** 10.3390/ma15238336

**Published:** 2022-11-23

**Authors:** Yujun Hu, Hongjin Zhao, Junwei Li, Kefu Hu, Jing Qin

**Affiliations:** 1 Faculty of Materials Metallurgy and Chemistry, Jiangxi University of Science and Technology, Ganzhou 341000, China; 2 School of Aeronautical Engineering, Jiangxi Teachers College, Yingtan 335000, China; 3 Guixi Junda Special Copper Materials Co., Ltd., Yingtan 335000, China; 4 College of Mechanical Engineering, Tongling University, Tongling 244000, China

**Keywords:** magnetic field treatments, aluminium bronze, dislocation density, grain boundaries, microhardness

## Abstract

The effect of a pulsed magnetic field on the microstructure of a QAl9-4 aluminium bronze alloy was studied in this work. It was found that the dislocation density, grain boundary angle, and microhardness of the alloy significantly changed after the magnetic field treatment with a peak magnetic induction intensity of 3T, pulse duration of about 100 us, pulse interval of 10 s, and pulse time of 360. EBSD was used to test the KAM maps of the alloy microzone. It was found that the alloy’s dislocation density decreased by 10.88% after the pulsed magnetic field treatment; in particular, the dislocation in the deformed grains decreased significantly. The quantity of dislocation pile-up and the degree of distortion around the dislocation were reduced, which decreased the residual compressive stress on the alloy. Dislocation motion caused LAGB rotation, which reduced the misorientation of adjacent points inside the grain. The magnetic field induced the disappearance of deformation twins and weakened the strengthening effect of twins. The microhardness test results show that the alloy’s microhardness decreased by 8.06% after pulsed magnetic field treatment. The possible reasons for the magnetic field effect on dislocation were briefly discussed. The pulsed magnetic field might have caused the transition to the electronic energy state at the site of dislocation pinning, which led to free movement of the vacancy or impurity atom. The dislocation was easier to depin under the action of internal stress in the alloy, changing the dislocation distribution and alloy microstructure.

## 1. Introduction

Aluminium bronze alloys are widely used in high-speed rail, nuclear power, ships, and other fields because of their excellent properties, such as high strength, wear resistance, and corrosion resistance [1,2]. With the rapid development of the high-speed railways, nuclear power generation, ocean engineering, and other industries, the properties of materials used in tooling equipment must meet increasingly stringent requirements. The development of high-performance aluminium bronze materials to meet the requirements of more complex working conditions has become a research hotspot. In recent years, researchers have successfully improved the related properties of aluminium bronze alloys utilizing plastic machining [3], heat treatment [4], ultrasonic vibration [5], additive manufacturing [6], and other techniques. However, among these technologies, pulsed magnetic field processing has been favoured by researchers because of its “non-contact processing,” ability, and it can improve the metal’s structure and properties without affecting the shape and appearance of the metal.

Researchers have tried to directly put metal materials in a strong magnetic field to study the impact of magnetic field treatment on material structure and properties. It was found that magnetic field treatment had apparent effects on improving the strength, plasticity, hardness, wear resistance, fatigue resistance, and many other properties of metal materials [7]. Reference [8] observed that after constant magnetic field treatment of 7055 aluminium alloy, the cellular dislocation transformed to network dislocation to form a subcrystal, which positively affected grain refinement, and η(MgZn_2_) transformed to the η′ phase. The tensile strength, elongation, and residual stress of the treated sample were reduced by 1.8%, increased by 40%, and decreased by 68.9%, respectively, compared with the untreated samples. Reference [9] found that when cold-rolled nickel–aluminium bronze NAB and extruded AA2014-T6 aluminium alloy were treated with an alternating magnetic field with a magnetic induction intensity of 1.24 T, many fine κ_Ⅳ_ phases precipitated in the NAB alloy and fine needle-like θ″ phases with a uniform distribution precipitated in the aluminium alloy. Compared with those that did not undergo magnetic field treatment, the microhardness of the NAB and aluminium alloy was increased by 6.2% and 4.5%, and the wear rate was reduced by 61% and 56%, respectively. Reference [10] reported that when cold-rolled EN8 steel and extruded AA2014-T6 aluminium alloy were also subjected to an alternating magnetic field with a magnetic induction intensity of 0.54 T, their respective fatigue lives were increased by 577% and 605%. Reference [11] investigated the effects of pulsed magnetic field treatment on a nickel-based alloy die and discovered that many fine γ′ phases precipitated in the γ matrix, the dislocation density increased, and the dislocation distribution became more uniform. Meanwhile, the average service life of the die was increased by 34.9%, and the tensile strength and elongation of the alloy increased by 7.3% and 34.6%, respectively.

Based on the advantages of magnetic field treatment, this work applied a strong pulsed magnetic field to a solid QAl9-4 aluminium bronze alloy for a high-speed rail brake system at room temperature. The changes in alloy microhardness before and after magnetic field treatment were tested, and micro-examinations were performed on the same microzone of the untreated and treated samples. The work aims to investigate the influence of the mechanism of the magnetic field on the alloy’s microstructure, further develop the magnetoplastic theory, and expand the engineering applications of the magnetic field. Meanwhile, the work also has value as a reference for improving the performance of QAl9-4 alloy parts with complex shapes.

## 2. Materials and Methods

**Figure 1:**
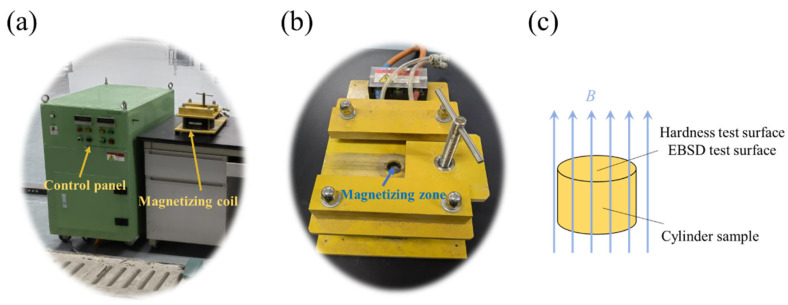
Experimental setup of pulsed magnetic field. (**a**) Magnetic treatment experiment, (**b**) magnetizing coil, (**c**) schematic diagram of sample treated with magnetic field.

The test material was a QAl9-4 aluminium bronze extruded rod used in manufacturing high-speed rail brake calipers. The chemical composition of the sample was 8.31 wt.% Al and 2.88 wt.% Fe, and the balance was Cu. The extrusion rod was processed into a Φ10 mm × 10 mm cylindrical sample by an electric spark wire-cutting machine and placed in EX-1520-30 pulsed magnetic field equipment for treatment. The output voltage was adjusted to 900 V; the peak magnetic induction intensity was about 3 T, and the duration of each pulse was about 100 us. The pulse interval was set to 10 s, and the sample was treated with 360 pulses. The magnetic field direction was parallel to the axial direction of the sample. A schematic diagram of the magnetic field treatment sample is shown in Figure 1. Then, the cross-sections of the sample before and after pulsed magnetic field treatment were processed for microhardness testing and microstructure observation. The 200HVS-5 Vickers hardness tester measured the microhardness of the sample with a load of 9.8 N and a holding time of 15 s. The grain boundary angle and dislocation density of the sample were analysed with a Zeiss-sigma scanning electron microscope with an HKL Technology Electron backscatter diffraction (EBSD) system.

## 3. Results

### 3.1. Effect of Magnetic Field Treatment on Dislocation

**Figure 2:**
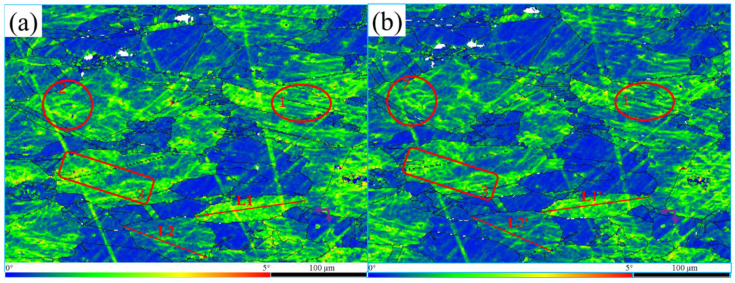
The KAM maps were collected for the same area of QAl9-4 aluminium bronze alloy before (**a**) and after (**b**) being treated by pulsed magnetic field. After magnetic field treatment, the KAM values in the red-highlighted regions 1, 2, and 3 showed a decreasing trend of dislocation density, and the KAM value indicated by the purple arrow showed a rising dislocation density trend. L1 and L2 were used to analyse the variation in point-to-point misorientation along the two lines, and the statistical results are shown in Figure 3.

The degree of plastic deformation or dislocation density increases as kernel average misorientation (KAM) value increases [12,13]. To study the alloy’s microscopic deformation and dislocation density, the degree of homogenization of the material’s plastic deformation was characterized by KAM. An EBSD test was used to obtain the KAM maps of the sample, as shown in Figure 2.

In the KAM map, the variation in dislocation density can be observed in both untreated and treated samples. As seen in Figure 2, the blue region had a lower KAM value, while the red region had a higher KAM value. By observing the colour distribution and changes in the KAM map, and analysing the dislocation distribution in each region, it can be determined that the yellow and green areas in the KAM map of samples treated were slightly reduced, while the blue areas were increased relative to untreated samples. Especially in regions 1, 2, and 3, marked in red in Figure 2, the blue area increased significantly, which indicates that the alloy’s dislocation density had a downward trend after magnetic field treatment. However, the KAM values, as indicated by the purple arrow, increased, indicating a tendency for the dislocation density to increase in the local region. The KAM value of the large area decreased, while that of the small area increased, indicating that the microscopic deformation degree of the alloy weakened, and the dislocation density tended to be more uniform after magnetic field treatment.

To more accurately analyse the variation in dislocation density, the following calculation formula was adopted according to strain gradient theory [14,15]:

$$\rho^{GND}=\frac{2KAM_{ave}}{\mu b}$$

where $\rho^{GND}$ is the mean geometrically necessary dislocations, *μ* is the unit length in the EBSD scanning step, *b* is the magnitude of the Burgers vector, and $KAM_{ave}$ represents the average misorientation of the selected area. In this experiment, *μ* was 0.2 μm, aluminium bronze *b* was 0.256 nm, and the $KAM_{ave}$ values of the untreated and treated samples were 0.016946699 and 0.015102888, respectively. According to the above formula, the dislocation densities of untreated and treated samples were 6.619 × 1014/m^2^ and 5.899 × 1014/m^2^, respectively. The dislocation density decreased by 10.88% after magnetic field treatment. As a result of the magnetic field treatment, the alloy may have experienced dislocation movement, annihilation, and proliferation, as evidenced by the drop in dislocation density and the tendency of dislocation distribution to homogenize.

**Figure 3:**
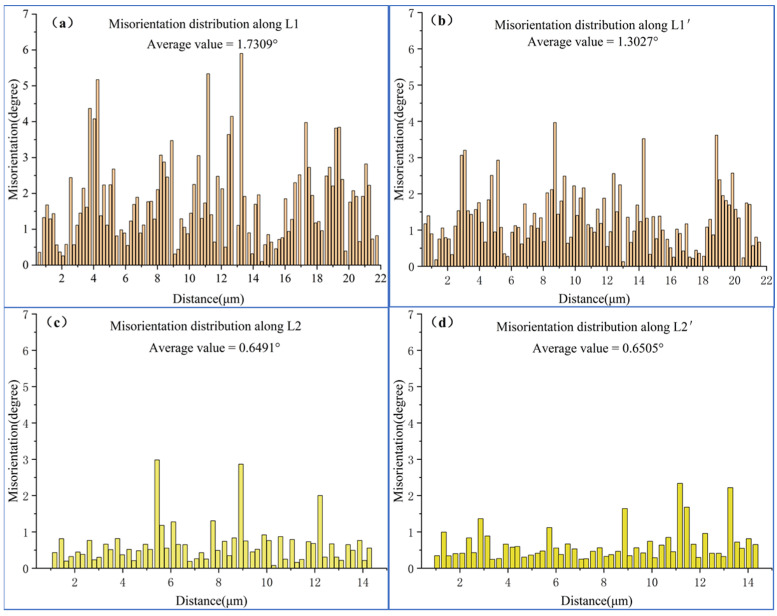
The misorientation distribution along the two lines in Figure 2 was collected before and after pulsed magnetic field treatment. (**a**)L1, (**b**) L1′, (**c**) L2, (**d**) L2′.

Adjacent grains with different dislocation densities, as listed in Figure 2, were chosen to further investigate the mechanism of the magnetic field effect on dislocation. The grain with line L1 had a high micro-deformation and dislocation density. The grain of line L2 had a low micro-deformation and dislocation density. The change in point-to-point misorientation on line segments L1 and L2 before and after magnetic field treatment was analysed, as shown in Figure 3. The average misorientation of L1 was 2.66 times higher than that of L2, indicating that the internal microscopic strains of the two grains were quite different. However, the average misorientation of L1′ was 2.00 times that of L2′, indicating that the difference in internal microscopic strain decreased after the magnetic field treatment. Moreover, misorientation decreased mainly due to the change from L1 to L1′. After magnetic field treatment, the average misorientation between L1 and L1′ decreased by 24.73%, while average misorientation between L2 and L2′ remained unchanged. The above results show that the decrease in dislocation density after magnetic field treatment was mainly due to the reduction in dislocation density in the grains with significant microscopic strain, which prompted the deformation among grains to become more uniform.

### 3.2. Effect of Magnetic Field Treatment on Grain Boundary

**Figure 4:**
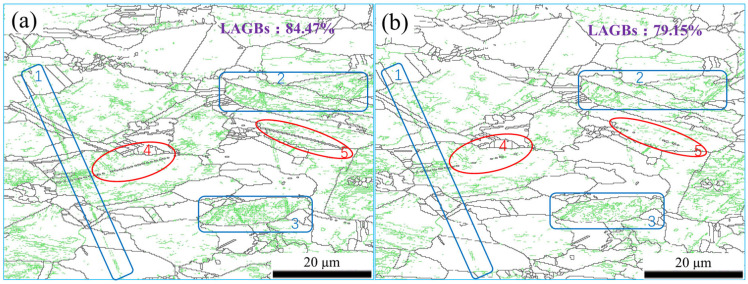
The grain boundary map was collected for the same area of QAl9-4 aluminium bronze alloy before (**a**) and after (**b**) being treated by pulsed magnetic field. The green lines represent LAGBs (less than 10°), but the black lines indicate LAGBs (more than 10°). Regions 1, 2, and 3 in blue displayed a notable decrease in LAGBs. In red regions 4 and 5, not only did the LAGBs decrease; HAGBs decreased even more severely.

The dislocation density decreased after magnetic field treatment, indicating that the dislocation shifted. When dislocations encountered obstacles, such as impurities and grain boundaries during movement, the pinning effect would occur, and stress concentration would form at the pinning place. If the stress concentration reached a certain degree, the dislocation would break through the energy barrier of the obstacle and cross the grain boundary [16]. The grain boundary angle could be changed by the behaviour of dislocation pile-up and crossing at the grain boundary. Figure 4 shows the grain boundary distribution of the sample. The green lines represent low-angle grain boundaries (LAGBs) with orientations less than 10°, while the black lines represent high-angle grain boundaries (HAGBs) with orientations greater than 10°. Statistical analysis of the whole observed area revealed that the proportion of LAGBs in the untreated sample was 80.47% and that in the treated sample was 78.53%. The number of LAGBs after magnetic field treatment was reduced. Further analysis of Figure 4 showed that the green lines in the blue boxes of regions 1, 2, and 3 were reduced significantly, indicating that the magnetic field promoted the disappearance of LAGBs. In the red circle regions 4 and 5, the black lines were reduced significantly more than the green lines, which indicates that the magnetic field affected not only the LAGBs, but also the HAGBs in this region. According to misorientation analysis of the HAGBs in regions 4 and 5, there was a nearly 60° misorientation between the two ends of the grain boundaries. It was determined that the grain boundaries were twin boundaries. Some twin boundaries deviated by approximately 6°, indicating that the twins deviated from the standard twin orientation due to the large plastic deformation during their formation [17]. The microstrain in the region where these twins were discovered was observed to be high, as was the KAM distribution in Figure 2, allowing the conclusion that these twins were deformation twins produced during alloy extrusion. In short, the application of a magnetic field could cause these deformation twins to vanish.

### 3.3. Effect of Magnetic Field Treatment on Microhardness

**Figure 5:**
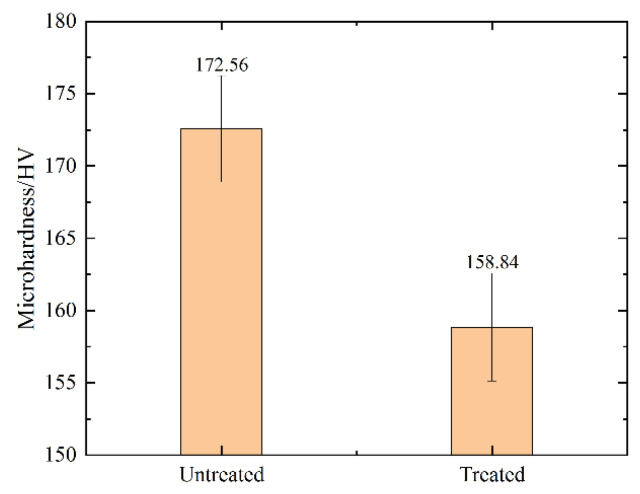
The microhardness of alloys before and after treatment.

Changes in the alloy’s microstructure, such as a drop in dislocation density, a reduction in the LAGBs, or the disappearance of deformation twins, will invariably affect its macroscopic properties. To avoid the effect of the sample’s uneven hardness on the test results, seven microhardness tests were conducted in the sample’s cross-section at a circumference location with a 5 mm diameter before and after the magnetic field treatment. The microhardness of the alloy changed before and after magnetic field treatment, as shown in Figure 5. It was discovered that the untreated sample’s microhardness value was 172.56 HV and its treated microhardness value was 158.84 HV. The magnetic field treatment caused an 8.06% decrease in microhardness. The amount of dislocation pile-up in the untreated samples varied after magnetic field treatment, which was the primary reason for the drop in the microhardness of samples. The application of a magnetic field accelerated the dislocation motion, resulting in the dislocation release from the obstacle and a reduction in the quantity of dislocation pile-up. As a result, the stress surrounding the dislocation was reduced, which changed the residual stress on a macro-level. The residual stress was closely linked with the alloy’s hardness. The alloy’s hardness increased with increasing residual compressive stress, while decreasing with increasing residual tensile stress [18,19]. Therefore, in the process of magnetic field treatment, the degree of dislocation pile-up and the level of distortion around the dislocation were reduced, which reduced the residual compressive stress on the surface of the alloy and led to a decrease in the microhardness of the material.

## 4. Discussion

According to the Frank–Read dislocation proliferation mechanism [20], when the tension $\tau_{T}$ acting on the dislocation line reaches the critical shear stress $\tau_{C}$ of the starting dislocation source, the dislocation starts to move. The critical shear stress $\tau_{C}$ can be expressed as:

(1) $$\tau_{T}\geq \tau_{C}=\frac{Gb}{L}$$

where *G* is the shear modulus, *b* is the burgess vector, and *L* is the dislocation line length. Generally, *L* ≈ 10^−6^ m and *b* ≈ 10^−10^ m are taken, which means that the critical shear stress of the starting dislocation source is $\tau_{C}$ ≈ 10^−4^*G*. The shear modulus *G* of the aluminium bronze alloy was 10^4^ MPa; thus, the $\tau_{C}$ of aluminium bronze alloy was no more than 10 MPa.

The residual stress of aluminium bronze alloy without magnetic field treatment was −96.1 MPa, measured by the sin2ψ method [21]. In terms of the order of magnitude of the tension exerted on the dislocation line, the residual stress inside the material can provide the driving force. However, the alloy’s impurity atoms, vacancies, and grain boundaries will pin and dissever the dislocation, and the dislocation line L will decrease sharply. According to Equation (1), the critical shear stress will increase rapidly, disenabling the movement of the dislocation. Therefore, the dislocation density of the aluminium bronze alloy decreased after pulsed magnetic field treatment. It is very likely that the magnetic field promotes dislocation depinning and improves dislocation depinning efficiency. In this way, the dislocation moves under the action of the original stress field and generates a magnetoplastic effect.

**Figure 6:**
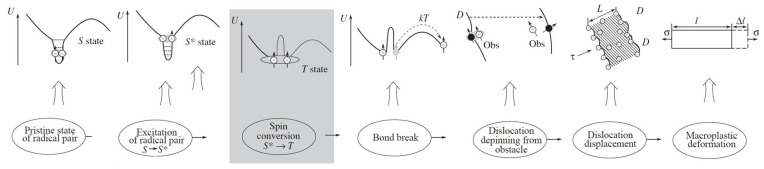
Schematic diagram of acceleration of dislocation depinning due to a magnetic field [25].

The mechanism by which the magnetic field promotes dislocation depinning is mainly free radical energy state conversion. Molotskii [22,23] pointed out that when the distance between the paramagnetic obstacle and the dislocation is less than a nanometre, the interaction between the paramagnetic obstacle and the dislocation will excite free electrons, resulting in the formation of a free radical pair between the paramagnetic obstacle and the dislocation. Free radical pairs can be classified into singlet states (S states) and triplet states (T states) by following the principles of quantum theory. The dislocation is tightly linked to the paramagnetic obstacle in the S state because the electron spins are antiparallel, and the binding bond is strong. As a result, the dislocation requires much energy to pass the obstacle. When the electron spins are parallel, the binding bond is weak in the T state. Thus, there is less binding energy between the dislocation and the obstacle, which reduces the amount of energy needed for the dislocation to pass the obstruction and keep moving. The dislocation can easily pass the obstruction in this way. The free radical pair frequently transitions between the S and T states when a magnetic field is present [23,24]. As soon as the free radical pair transitions into the T state, the system’s energy increases, the stability decreases, and the dislocation is more accessible to depin and move. It has been proved that paramagnetic obstructions can be observed in any crystal, such as metal, semiconductor, and dielectric materials, at any temperature [23]. Golovin also used magnetic field to change the electron spin state to explain how the magnetic field promoted the behaviour of dislocation unpinning in ionic crystals, and provided a schematic diagram of magnetic-field-enhanced dislocation unpinning, as shown in Figure 6 [25]. Since both metallic and covalent bonds result from the interaction between electrons and atoms, this diagram is helpful to understand how magnetic fields accelerate dislocation depinning in metals.

According to the above theory, it can be inferred that the dislocation in the aluminium bronze alloy becomes more easily depinned and moved under the influence of the original stress field, due to the change in the electronic energy state at the pinned place during the pulsed magnetic field treatment. On the one hand, the dislocation movement reduced the stress concentration caused by the dislocation pile-up, relaxed the original stress, and reduced the residual stress of the alloy, as verified by the significant reduction in residual stress in the magnetic field treatment of aluminium alloy [7], nickel–aluminium bronze [9], EN8 special steel [10], titanium alloy [26], and magnesium alloy [27]. On the other hand, the dislocation movement somewhat altered the grain boundary angle, resulting in an apparent reduction in LAGBs and the disappearance of twin boundaries, which lowered the system energy.

As shown in Figure 4a, the proportion of LAGBs in the extruded aluminium bronze alloy was as high as 80.47%. Many LAGBs evolved from dislocation walls formed by dislocation accumulation and rearrangement during extrusion. The density of LAGBs also reflected the dislocation density of the deformation structure. As shown in Figure 4b, the proportion of LAGBs in alloy treated with a magnetic field decreased to 78.53%. LAGBs were composed of a series of dislocation arrays, which had a weak hindering effect on dislocation movement. The evolution of LAGBs largely depends on dislocation movement [28,29]. After magnetic field treatment, the dislocation movement became more flexible, resulting in dislocation rearrangement and annihilation, and dislocation density decreased. According to Chen Y et al. [30], grain rotation was the dominant mechanism of grain growth when there was a slight variation in grain size and misorientation, as well as a high degree of symmetry in the grain’s structure. In Figure 4, the maximum value of point-to-point misorientation along L1 was within 6°. Based on this, it can be inferred that the rearrangement of dislocations leads to the rotation of LAGBs and the gradual transformation to HAGBs.

A twin boundary is a special kind of HAGB. In the deformation process, the layer-by-layer superposition of Shockley partial dislocations generates deformation twins, and the interaction between the twin boundary and dislocations induces the detwinning behaviour [31,32]. Under the action of the magnetic field, the mobility of dislocation was enhanced, and the slip of partial dislocations on the twin boundary led to the occurrence of detwinning and the disappearance of deformation twins. The disappearance of twins weakened their strengthening effect on the alloy, which was mutually verified by the decrease in the alloy’s microhardness.

**Figure 7:**
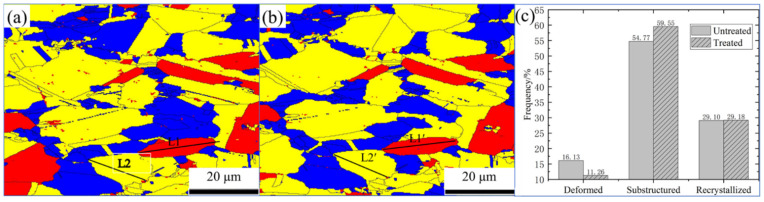
The grain distribution map was collected in the same area of QAl9-4 aluminium bronze alloy before (**a**) and after (**b**) being treated by pulsed magnetic field. (**c**) Grain content statistics. Yellow denotes substructure, red denotes deformed grains, and blue denotes recrystallized grains.

The grain’s distribution is shown in Figure 7, where yellow denotes the substructure, red denotes the deformed grains, and blue denotes the recrystallized grains. It was discovered that following magnetic field treatment, the proportion of the substructure grew from 54.77% to 59.55%, the deformed grain was reduced from 16.13% to 11.26%, and the recrystallized grains essentially remained unchanged. This reveals that the magnetic field obviously affected the deformed grains. This was consistent with the fact that the average point-to-point misorientation along L1 to L1′ decreased by 24.73%, as seen in Figure 4, while the average point-to-point misorientation along L2 to L2′ basically remained unchanged. As a result of their greater sensitivity to magnetic fields, the deformation grains showed that the effect of magnetic fields on alloys was mainly due to an increase in the efficiency of dislocation depinning and rearrangement in the deformation grains. Dislocations were eliminated during the rearrangement process, resulting in a reduction in dislocation density and the rotation of LAGBs. This enhances the release of internal deformation energy storage and increases the stability of the alloy system.

## 5. Conclusions

(1) The pulsed magnetic field treatment could change the distribution of dislocations and eliminate dislocations in the aluminium bronze alloy, decreasing dislocation density, especially in deformed grains.
(2) The pulsed magnetic field can improve the efficiency of dislocation depinning of aluminium bronze alloy. The dislocation moves under the action of the magnetic field and original stress field, which causes the rotation of LAGBs. The misorientation of adjacent points inside the grain gradually decreases.
(3) The magnetic field decreases the alloy’s residual stress and the quantity of dislocation pile-up. Meanwhile, the magnetic field induces the elimination of deformation twins and mitigates the strengthening effect of twins. Macroscopically, it demonstrates a decrease in the alloy’s microhardness.

## Data Availability

Not applicable.
